# Comparative investigation on the sizes and scavenger receptor binding of human native and modified lipoprotein particles with atomic force microscopy

**DOI:** 10.1186/s12951-018-0352-3

**Published:** 2018-03-21

**Authors:** Chaoye Gan, Kun Wang, Qisheng Tang, Yong Chen

**Affiliations:** 1 Nanoscale Science and Technology Laboratory, Institute for Advanced Study, Nanchang University, 999 Xuefu Ave., Honggutan District, Nanchang, 330031 Jiangxi China; 2 College of Life Sciences, Nanchang University, Nanchang, 330031 Jiangxi China

**Keywords:** Lipoproteins, Atomic force microscopy (AFM), Scavenger receptors, CD36, SR-B1

## Abstract

**Background:**

The size and receptor-binding abilities of plasma lipoproteins are closely related with their structure/functions. Presently, the sizes of native lipoproteins have been measured by various methods including atomic force microscopy (AFM) whereas the sizes of modified lipoproteins are poorly determined and the receptor-binding ability of lipoproteins is less detected and compared at the nanoscale.

**Methods:**

Here, AFM was utilized to detect/compare the size and scavenger receptor-binding properties of three native human lipoproteins including high-density lipoprotein, low-density lipoprotein (LDL), and very low-density lipoprotein, and two modified human lipoproteins including oxidized and acetylated LDL, as well as bovine serum albumin and their antibodies as negative and positive controls, respectively.

**Results:**

AFM detected that the sizes of these lipoproteins are close to the commonly known values and the previously-reported AFM-detected sizes and that native and modified LDL have different height/size. AFM also revealed that the CD36-binding abilities of the five lipoproteins are different from one another and from their SR-B1-binding abilities and that the anti-CD36/SR-B1 antibodies as positive controls have strong CD36/SR-B1-binding abilities.

**Conclusions:**

The data provide important information on lipoproteins for better understanding their structures/functions. Moreover, the data certify that besides size measurement AFM also can visualize receptor-lipoprotein binding at the nanoscale, as well as antigen–antibody (scavenger receptors and their antibodies) binding.

## Background

Plasma lipoproteins, e.g. native low-density lipoprotein (LDL), very low-density lipoprotein (VLDL), high-density lipoprotein (HDL), among others, play vital roles in transport and delivery of lipids, including cholesterol, triglycerides, and some lipid-soluble vitamins, to or from peripheral tissue cells. The lipoproteins, particularly their modified forms, e.g. oxidized LDL (oxLDL) and acetylated LDL (acLDL), have been associated with the pathophysiology of many disease states, including diabetes, atherosclerosis, cancer, and others [1]. Therefore, it is meaningful and significant to investigate the structural and receptor-binding properties of these lipoproteins for better understanding their physiological functions or roles in diseases.

At present, multiple approaches are available to investigate the structural and receptor-binding properties of plasma lipoproteins, such as dynamic light scattering (DLS), nuclear magnetic resonance (NMR), cryo-electron microscopy (cryo-EM), etc. for detection of the structural properties, and immunoassay, fluorescence methods, surface plasmon resonance (SPR), etc. for detection of the receptor-binding properties. Currently, however, there are no ideal tools capable of detecting or even “seeing” both the structural and receptor-binding properties of plasma lipoproteins particularly under a physiological condition. Atomic force microscopy (AFM) may be a candidate of such tool.

After decades of development and applications, AFM has proved to be a promising and powerful technique for nanoscale detection of biological samples with a broad size range from micro-sized tissues/cells down to nano-sized molecules [2–6]. AFM can not only image individual biological molecules at the nanoscale but also directly “see” molecule–molecule (e.g. biotin-streptavidin, antigen–antibody, ligand-receptor, etc.) binding/interaction [7–9] also at the nanoscale even under physiological conditions.

Surprisingly, however, the AFM studies on plasma/circulating lipoproteins, an important type of natural lipid-protein complexes with a nanoscale size (generally 5–80 nm; chylomicrons have a size of up to 1 μm), are limited to the imaging and/or size measurement of native lipoproteins (mainly LDL and HDL) [10–17], and even no AFM size measurement of modified lipoproteins (e.g. oxLDL or acLDL) was previously reported until now. Besides the size, the receptor-binding property is also correlated tightly with the structure and functions of lipoproteins. Unfortunately, however, AFM studies on this property of lipoproteins are also missing.

In this study, we utilized the powerful imaging function of AFM to investigate the sizes of multiple native/modified lipoproteins including HDL, LDL, oxLDL, acLDL, and VLDL. Thereafter, the abilities of these lipoproteins binding to CD36 or SR-B1 (two major type B scavenger receptors) were evaluated by AFM at the nanoscale. The data showed that AFM is able to detect/“see” both the structural and receptor-binding properties of plasma lipoproteins at the nanoscale under physiological conditions (e.g. in PBS).

## Methods

### Reagents

Human LDL, oxLDL, acLDL, and HDL were purchased from Yiyuan Biotechnologies (Guangzhou, China). Human VLDL was from American Research Products (MA, USA). All lipoproteins were used immediately and stored at 4 °C for at most 3 weeks. Recombinant human CD36 and SR-B1, as well as anti-CD36 and anti-SR-B1 antibodies, were purchased from Abcam (Cambridge, MA). *N*,*N*-Diisopropylethylamine (DIPEA), aminopropyltriethoxysilane (APTES), and glutaraldehyde were from TCI (Shanghai, China; or Tokyo, Japan for APTES). Bovine serum albumin (BSA) was from Solarbio (Beijing, China).

### Mica functionalization and sample preparation

The method for mica functionalization and sample preparation was modified from previous reports [16–18]. The first part of the procedure was performed in a clean and dry glass dessicator (2.5 l capacity, no dessicants, with a walve in the cover). Ultra pure argon was used to remove the air and moisture (~ 2 min) in the dessicator. The freshly cleaved mica sheets in clean petri dishes were immediately put inside following with argon purging again for ~ 2 min. Next, 30 μl of DIPEA and 50 μl of APTES were carefully pipetted into two containers in the dessicator. After argon purging for 0.5–1 min, the mica sheets were exposed to APTES vapor for ~ 2 h. After the APTES container was carefully removed, the dessicator was purged with argon and sealed, and the treated mica sheets were stored inside. Two days later, the mica sheets were removed from the dessicator and immediately incubated with 100 μl of 0.2% fresh glutaraldehyde solution in double distilled water for 10 min. After the mica sheets were rinsed with double distilled water, protein/lipoprotein samples were deposited immediately for 2 h, washed with PBS, incubated with l-glycine for ~ 15 min, washed again, and then subjected to AFM detection. For lipoprotein-receptor binding experiments, the functionalized mica sheets were rinsed, incubated with receptors (100 μl CD36 or SR-B1 at 0.1 μg/ml using BSA as a control) or heat-inactivated receptors/BSA (as controls), washed twice, incubated with l-glycine, washed again, and subsequently incubated with 100 μl lipoproteins at 0.1 μg/ml. After washing with PBS to remove excess lipoproteins, the samples were subjected to AFM imaging, or the samples were stained with Oil Red O and rinsed, and then the mica sheets bearing the samples were imaged.

### AFM imaging

An Asylum MFP-3D-SA AFM (Asylum Research, USA) equipped with a scanner of 90 μm × 90 μm × 15 μm was utilized. AFM was performed in liquid (PBS) in tapping mode. The data were acquired using silicon nitride tips (AppNano, USA) with an end radius of 10 nm and a spring constant of ~ 0.04 N/m. To obtain topographical images, the AFM probe was scanned across the mica surface (at 0.5–1 Hz) with a tracking force of 300–500 pN. The data were processed using the instrument-equipped software (Igor Pro 6.31) and all images were flattened by one level. Using AFM topographical images, the height (*h*) and radius (*r* is half of the full width at half maximum (FWHM)) were measured/calculated. Then, the volume (*V*) of a single particle was calculated using Eq. 1 [19], based on which the equivalent diameter of a sphere was calculated.

1 $$ V\, = \,\left( {{{\pi \cdot h} \mathord{\left/ {\vphantom {{\pi \cdot h} 6}} \right. \kern-0pt} 6}} \right) \cdot \left( { 3r^{ 2} \, + \,h^{ 2} } \right) $$

### Statistical analysis

All data are from at least three independent experiments. Statistical analyses were performed using one-way ANOVA to determine the statistically significant differences between different groups. A value of *p *< 0.05 was considered statistically significant.

## Results and discussion

### AFM imaging and size measurement of various lipoproteins at the nanoscale

AFM has been utilized to image and measure majorly LDL and HDL whereas AFM imaging and size measurement of VLDL and particularly modified LDL (e.g. acLDL and oxLDL) were less performed. Here, five types of lipoproteins including LDL, HDL, VLDL, acLDL, and oxLDL were imaged/measured and compared by AFM at the same time.

**Fig. 1:**
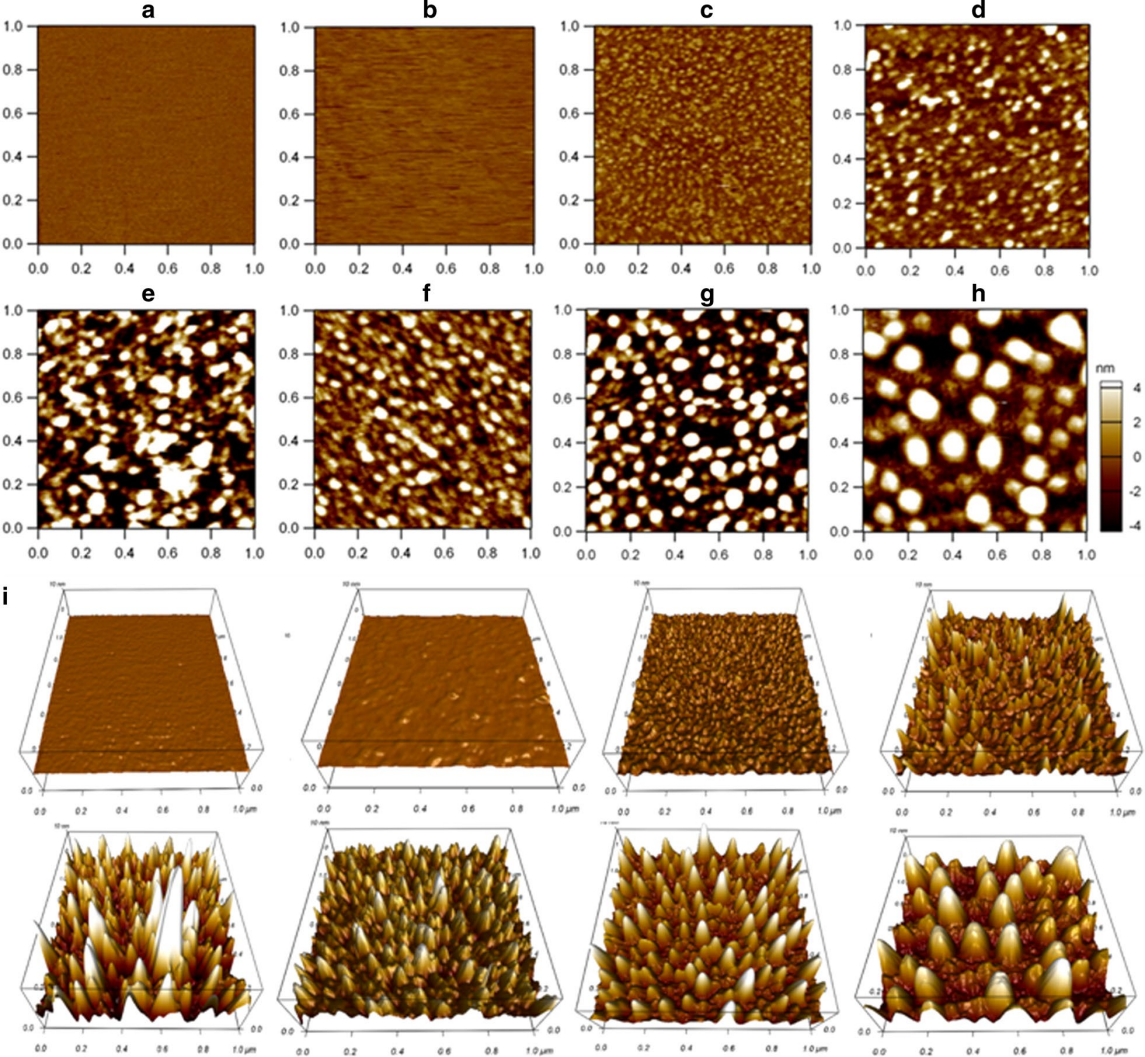
AFM topographical images of multiple lipoproteins. **a** Bare mica; **b** functionalized mica; **c** BSA; **d** HDL; **e** LDL; **f** oxLDL; **g** acLDL; **h** VLDL. Scan range: 1 μm × 1 μm. **i** The corresponding 3-dimensional images of **a**–**h**

**Fig. 2:**
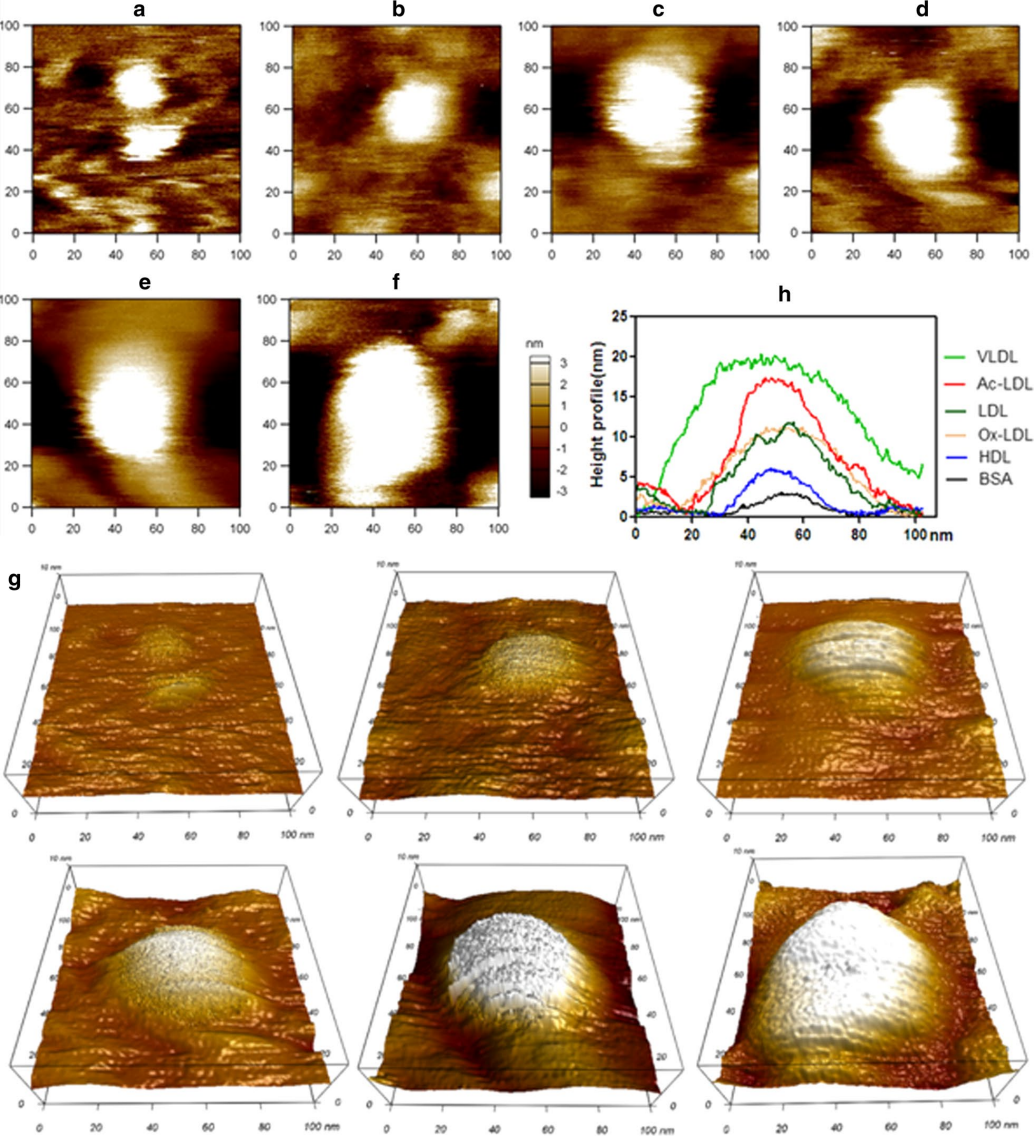
AFM topographical images of single lipoproteins. **a** BSA; **b** HDL; **c** LDL; **d** oxLDL; **e** ac-LDL; **f** VLDL. Scan range: 100 nm × 100 nm. **g** The corresponding 3-dimensional images of **a**–**f**. **h** Height profiles of these lipoproteins

Prior to lipoprotein detection, the bare mica and GD-APTES-mica were imaged by AFM in PBS (Fig. 1a, b). The surfaces of these micas were very smooth on which no particles were observed. Figure 1c–h shows the AFM topographical images (scan range: 1 μm × 1 μm) of BSA, HDL, LDL, oxLDL, acLDL, and VLDL, respectively. Figure 1i shows their corresponding 3-dimensional (3-D) images. Figure 2a–f shows the representative AFM topographical images (scan range: 100 nm × 100 nm) of single lipoprotein particles (BSA, HDL, LDL, oxLDL, acLDL, and VLDL, respectively) and Fig. 2g shows their corresponding 3-D images. It is well known that plasma lipoproteins are heterogeneous in size. Based on the size heterogeneity, plasma lipoproteins have generally been categorized into different subclasses. In this study, it is not surprise that the lipoprotein particles visualized by AFM were heterogeneous in size since each lipoprotein sample might contain all subclasses. Visually, However, the sizes are comparable among different lipoprotein types as follows: BSA<HDL<LDL/oxLDL/acLDL<VLDL (there are no obvious differences in particle size among LDL, oxLDL, and acLDL). Figure 2h displays the cross-section height profiles of these lipoprotein particles. It is evident that the comparison of the lateral dimension among these particles coincides with the topographical images. Interestingly, acLDL has a higher height profile than LDL. Further quantification and statistical analysis of the particle sizes (Table 1) show that the comparison of their equivalent diameters of a sphere is as follows: BSA<HDL<LDL/oxLDL<acLDL<VLDL. The equivalent diameters of a sphere with the calculated volume from the full width at half maximum (FWHM) are 8.4 ± 1.5, 11.5 ± 2.4, 19.5 ± 4.6, 19.1 ± 3.2, 21.8 ± 3.6, and 42.8 ± 7.1 nm for BSA, HDL, LDL, oxLDL, acLDL, and VLDL, respectively.

**Table 1:** AFM-measured sizes of BSA, lipoproteins, receptors and their antibodies

	Height (nm)	Diameter/FWHM (nm)	Equivalent diameter of a sphere (nm)
BSA	2.2 ± 0.4	18.9 ± 4.3	8.4 ± 1.5
HDL	4.0 ± 0.9	22.2 ± 5.7	11.5 ± 2.4
LDL	8.9 ± 2.4	31.8 ± 8.6	19.5 ± 4.6
Ox-LDL	7.5 ± 1.5	34.4 ± 7.0	19.1 ± 3.2
Ac-LDL	10.8 ± 2.9	34.0 ± 6.0	21.8 ± 3.6
VLDL	11.2 ± 2.2	95.9 ± 15.6	42.8 ± 7.1
CD36	2.6 ± 0.6	18.2 ± 4.5	8.6 ± 1.5
SR-B1	2.5 ± 0.5	18.4 ± 5.3	8.6 ± 1.7
Anti-CD36	3.0 ± 1.0	22.5 ± 3.9	10.5 ± 1.8
Anti-SR-B1	3.3 ± 0.8	20.8 ± 4.0	10.3 ± 1.7

n = ~ 255 for each group

The AFM-measured sizes of HDL, LDL, and VLDL in our study are close to the commonly known or previously reported AFM-measured values [10–16, 20, 21]. This result also implies that the individual particles imaged by AFM are single lipoproteins (not lipoprotein aggregates). The size relationship among native lipoproteins (i.e. HDL<LDL<VLDL) is well known. However, the size relationship between LDL and modified LDL (e.g. oxLDL and/or acLDL) remains unclear. For the first time, the sizes of LDL, oxLDL, and acLDL particles were compared here. Interestingly, the statistical analysis (Table 1) shows that when deposited on a substrate/mica oxLDL particles display a significantly smaller average height (7.5 ± 1.5 nm) than LDL particles (8.9 ± 2.4 nm) although they have a similar equivalent diameter of a sphere (19.1 ± 3.2 and 19.5 ± 4.6 nm for oxLDL and LDL, respectively; *p *> 0.05) whereas acLDL particles (height: 10.8 ± 2.9 nm) are significantly thicker and larger than LDL particles (the equivalent diameter of a sphere: 21.8 ± 3.6 and 19.5 ± 4.6 nm for acLDL and LDL, respectively; *p *< 0.001).

It is well known that oxidation can lead to loss of LDL lipids (generating biologically active products, e.g. peroxides, aldehydes, lyso-PC, oxysterols, etc.) and even hydrolysis/fragmentation of apoB-100 [22, 23] whereas acetylation generally modifies the amino acid residues (e.g. the ε-amino group of lysine) of LDL without alterations in lipid and protein composition of LDL [24]. Therefore, compared with native LDL, the oxidation-induced partial loss of LDL components might contribute to the decrease in size/height of oxLDL whereas the increase in size/height of acLDL might result from the addition of acetyl groups and/or increased hydrophobicity of the acetylated surface.

### AFM visualizes the receptor-lipoprotein binding at the nanoscale

**Fig. 3:**
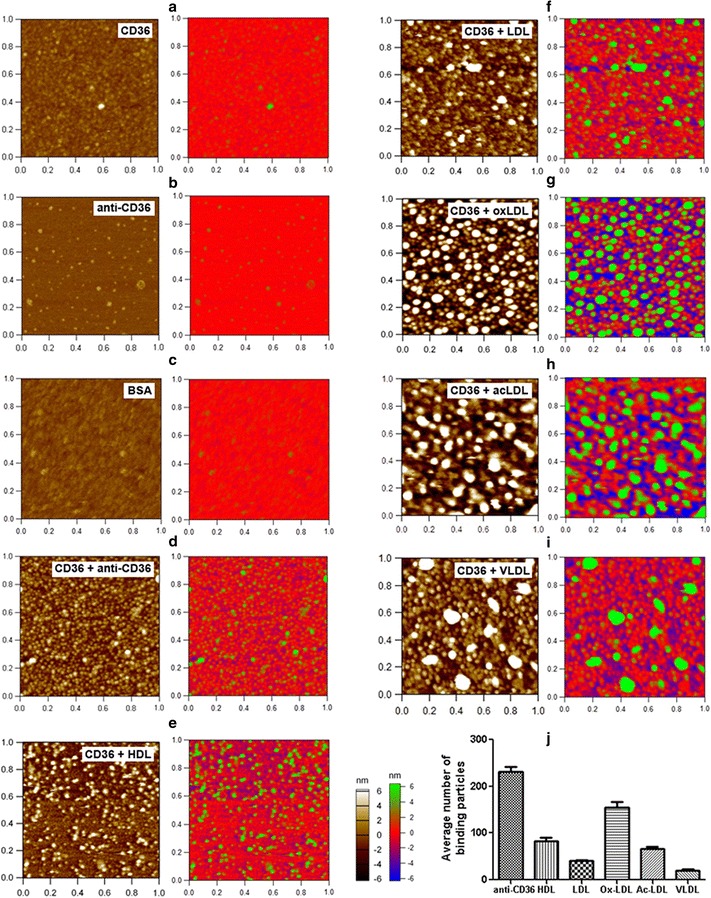
Direct visualization of CD36-binding abilities of various lipoproteins. **a**, **b** AFM topographical images of a CD36 monolayer (**a**) and anti-CD36 antibody particles (**b**); **c**–**i** AFM topographical images of BSA (**c**), anti-CD36 antibody (**d**), and lipoproteins (**e**–**i**: HDL, LDL, oxLDL, acLDL, and VLDL, respectively) binding to a CD36 monolayer. Scan range: 1 μm × 1 μm. Right panels: the images are showed in multicolor to clearly distinguish the binding proteins/lipoproteins (green) from the CD36 monolayer (red). **j** Quantification of the average number of CD36-binding antibody/lipoprotein particles in a scan range. *p *< 0.05 for all pairs of columns except for HDL vs. acLDL

**Fig. 4:**
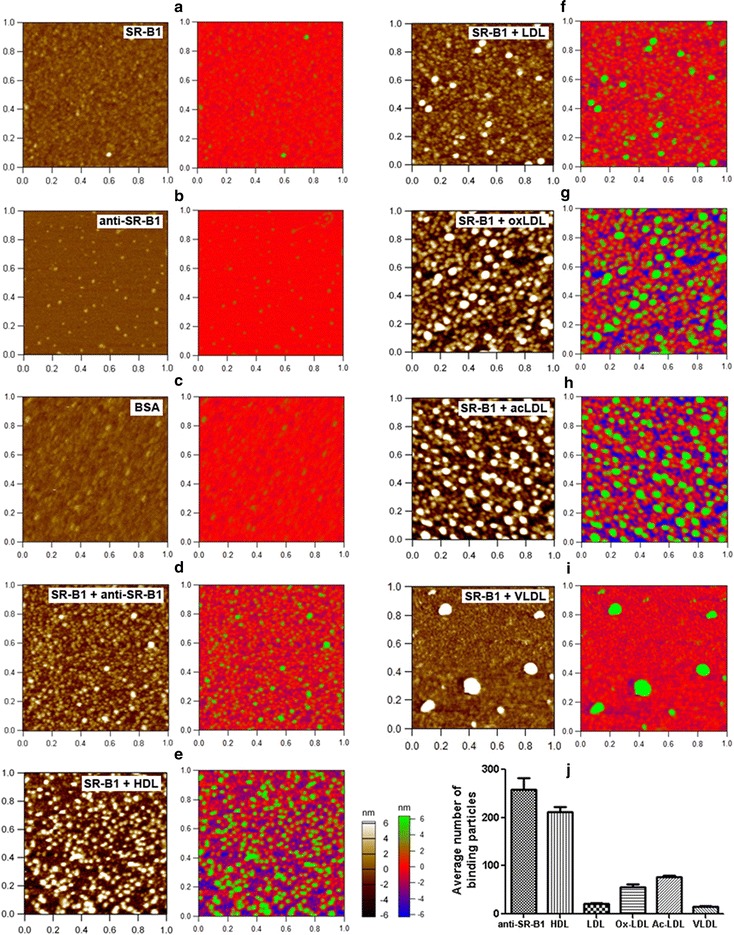
Direct visualization of SR-B1-binding abilities of various lipoproteins. **a**, **b** AFM topographical images of a SR-B1 monolayer (**a**) and anti-SR-B1 antibody particles (**b**); **c**–**i** AFM topographical images of BSA (**c**), anti-SR-B1 antibody (**d**), and lipoproteins (**e**–**i**: HDL, LDL, oxLDL, acLDL, and VLDL, respectively) binding to a SR-B1 monolayer. Scan range: 1 μm × 1 μm. Right panels: the images are showed in multicolor to clearly distinguish the binding proteins/lipoproteins (green) from the SR-B1 monolayer (red). **j** Quantification of the average number of SR-B1-binding antibody/lipoprotein particles in a scan range. *p *< 0.05 for all pairs of columns except for LDL vs. VLDL

Among more than dozen types of well-known scavenger receptors, CD36 and SR-B1 are the two receptors known to bind both native (e.g. HDL, LDL, VLDL) and modified (e.g. oxLDL and acLDL) lipoproteins [25, 26]. However, less comparison of the scavenger receptor-binding ability among the five lipoprotein types was quantitatively performed previously. Moreover, all the related previous knowledge was majorly obtained from cellular experiments generally using fluorescence microscopy and/or flow cytometry and the complicated environment of cells might make the data questionable. For instance, each type of lipoprotein may bind to different scavenger receptors or non-receptor molecules which potentially co-exist on the same cell; dynamic internalization/degradation of receptor-binding lipoproteins, differential expression levels of the same receptors induced by distinct lipoproteins, and others can occur on/in living cells. Here, we tested the possibility of utilizing AFM to directly visualize receptor-lipoprotein binding and compared the CD36/SR-B1-binding ability among the five types of lipoproteins at the nanoscale (Figs. 3, 4).

Prior to investigation on the lipoprotein-receptor binding, CD36 and SR-B1 (Figs. 3a, 4a), as well as their antibodies (Figs. 3b, 4b), on GD-APTES-mica were imaged by AFM in PBS. The quantification data (Table 1) shows that the height and equivalent diameter of CD36/SR-B1 particles are ~ 2.6 and ~ 8.6 nm, respectively (*p *> 0.05 for CD36 vs. SR-B1), and that the height and equivalent diameter of their antibodies are ~ 3.0 and ~ 10.4 nm, respectively (*p *> 0.05 for anti-CD36 vs. anti-SR-B1). For the first time, scavenger receptor molecules (CD36 and SR-B1) were imaged and size-measured by AFM.

It implies that CD36/SR-B1 particles are significantly more flat and smaller than lipoprotein particles of all five types (Table 1; *p *< 0.001 for CD36/SR-B1 vs. each of the five lipoprotein types), based on which lipoprotein particles can be distinguished from receptor particles in the same field for direct visualization and quantification of receptor-lipoprotein binding. Even the anti-CD36/anti-SR-B1 antibodies with similar size (Table 1) could be identified from the CD36/SR-B1 monolayer based on an increased height due to the stacking/binding of antibody molecules onto the receptor monolayer (left panels of Fig. 3d and left panels of Fig. 4d). These data imply that utilizing AFM to directly visualize receptor-lipoprotein binding at the nanoscale is feasible and reliable.

Next, CD36 or SR-B1 molecules immobilized on GD-APTES-mica were incubated with each of the five types of lipoproteins in PBS. After removal of unbound lipoproteins, the samples were imaged by AFM in PBS (left panels of Figs. 3e–i, 4e–i). Clearly, two layers of particles were visualized in AFM topographical images, the bottom receptor monolayer with small particles and the upper lipoprotein layer with significantly larger and brighter particles. To make the topographical images more intuitive, pseudocolors were used to highlight the upper antibody/lipoprotein layers (green) by adjusting the bottom receptor monolayer to the red baseline (the Right panels of Fig. 3a–i and right panels of Fig. 4a–i). The pseudocolor Z scale bar was obtained by adjusting the Z scale bar of the control group (only CD36/SR-B1 monolayer on mica) to the situation (all particles were invisible), then all groups used the same pseudocolor Z scale bar. The size of each of the five lipoprotein types in the upper layer coincides with that of each lipoprotein directly deposited on substrate (Fig. 1), implying the reliability of the data and that the individual particles in the upper layer are single lipoproteins.

Obviously, all five types of lipoproteins could bind to CD36 or SR-B1 but to different extents. As a negative control, no BSA particles were found to bind onto the receptor layers (Figs. 3c, 4c). As a positive control, a large quantity of antibody (anti-CD36 or anti-SR-B1) was detected on the receptor layers (Figs. 3d, 4d). Statistical analyses further confirmed the observations and revealed that the receptor-binding abilities of the five lipoproteins are as follows: oxLDL>HDL/acLDL>LDL>VLDL for CD36 binding (Fig. 3j) and HDL>acLDL>oxLDL>LDL/VLDL for SR-B1 binding (Fig. 4j). Since the size/height of antibodies is smaller than that of all lipoprotein types the number of receptor-binding antibody particles in the topographical images (Figs. 3d, 4d) may visually be less abundant than that of CD36-binding oxLDL (Fig. 3g) or SR-B1-binding HDL (Fig. 4e).

Among the five lipoprotein types, oxLDL has an extremely (> 2-fold) stronger CD36-binding ability (Fig. 3g, j) whereas HDL has an extremely (> 3-fold) stronger SR-B1-binding ability (Fig. 4e, j) than the other lipoproteins. The results are consistent with the original characterizations of CD36 and SR-B1 as lipoprotein receptors for oxLDL [27] and HDL [28, 29], respectively, and also consistent with the well-known major roles of CD36 and SR-B1. The results further certify the effectiveness and reliability of the method.

**Fig. 5:**
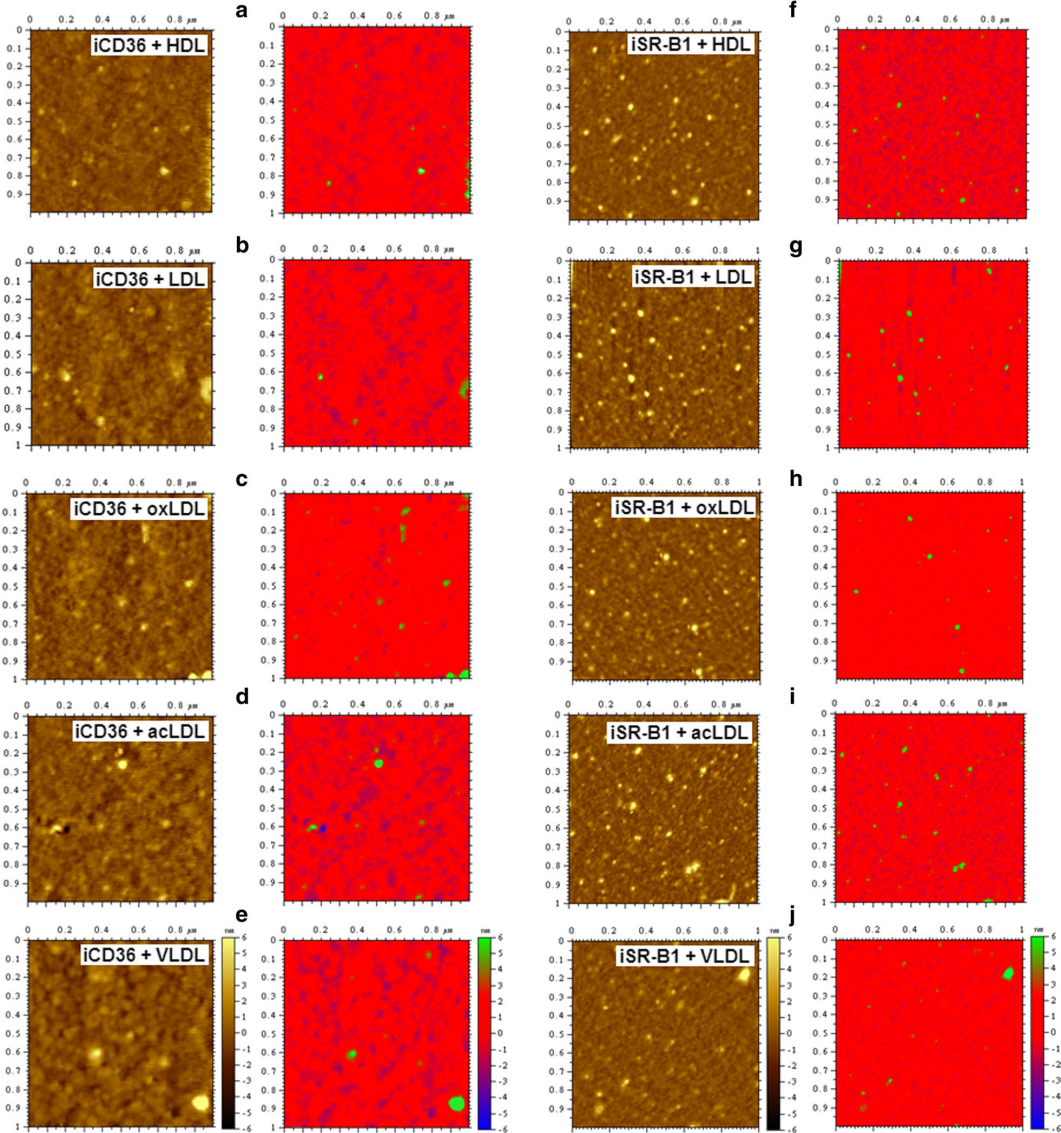
AFM topographical images of the samples using heat-inactivated CD36 (abbreviated as iCD36; **a**–**e** or heat-inactivated SR-B1 (abbreviated as iSR-B1; **f**–**j**) as the bottom monolayer and incubating with HDL (**a**, **f**), LDL (**b**, **g**), oxLDL (**c**, **h**), acLDL (**d**, **i**), and VLDL (**e**, **j**), respectively. Scan range: 1 μm × 1 μm. Right panels: the images are showed in multicolor to clearly distinguish the binding proteins/lipoproteins (green) from the CD36/SR-B1 monolayer (red)

**Fig. 6:**
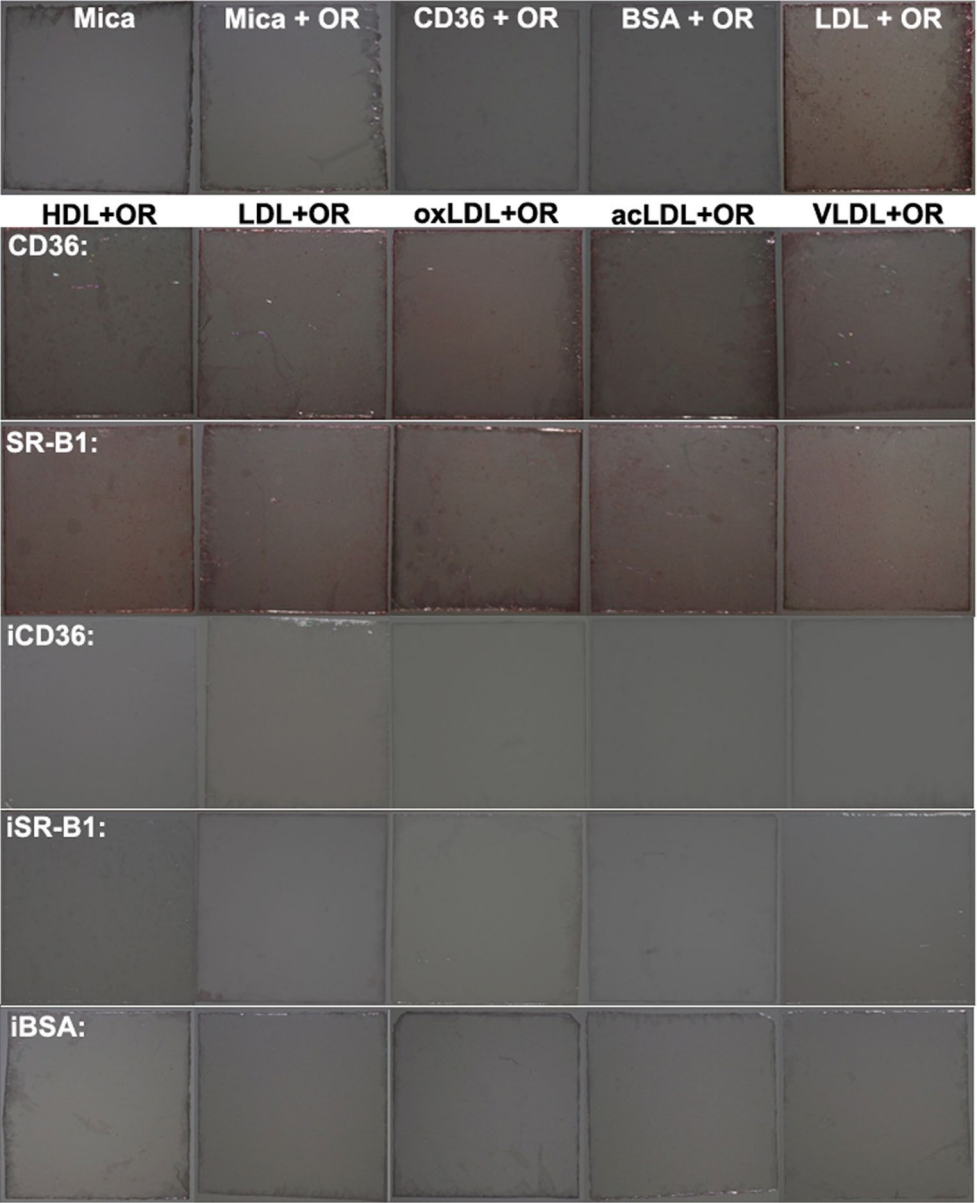
Observation of the whole mica sheets on which the samples were stained with Oil Red O (abbreviated as OR). The uppermost panel (from left to right): functionalized mica without OR staining; functionalized mica with OR staining; CD36 monolayer with OR staining; BSA monolayer with OR staining; LDL monolayer with OR staining. From the second panel to the bottom panel: the micas were coated with a monolayer of CD36, SR-B1, heat-inactivated CD36 (iCD36), heat-inactivated SR-B1 (iSR-B1), or heat-inactivated BSA (iBSA), respectively then incubated with the five lipoproteins (HDL, LDL, oxLDL, acLDL, and VLDL, respectively from left to right) and stained with Oil Red O (OR)

To test whether nonspecific binding might occur during the above experiments, two additional experiments were conducted. First, AFM imaging of lipoprotein-receptor binding was re-performed by only replacing the receptors (CD36 or SR-B1) with heat-inactivated receptors (abbreviated as iCD36 or iSR-B1) on the bottom monolayer. The data (Fig. 5) showed that no (or only a few) lipoprotein particles nonspecifically bound to the heat-inactivated CD36/SR-B1 monolayer. Second, instead of a tiny area (1 μm × 1 μm) of mica sheets imaged by AFM, the whole mica sheets (~ 5 mm × 5 mm) on which the samples were stained with Oil Red O (a widely used dye for lipids or lipid-containing particles, e.g. plasma lipoproteins) were observed and imaged by a camera (Fig. 6). The data showed that mica and protein molecules (e.g. CD36, BSA, etc.) could not be stained by Oil Red O (abbreviated as OR) whereas the LDL-bearing mica sheet was stained in red (the uppermost panel) and that all the mica sheets using CD36/SR-B1 (panels 2 and 3, respectively) but not heat-inactivated CD36/SR-B1/BSA (abbreviated as iCD36, iSR-B1, and iBSA, respectively; panels 4–6, respectively) as the bottom monolayer were in red. Both data confirmed that nonspecific binding did not happen significantly in the AFM study on lipoprotein-receptor binding.

For the first time, we determined the receptor-binding abilities of all five lipoprotein types. Unfortunately, we currently do not know the underlying mechanisms of causing the differences in receptor-binding ability among various lipoproteins. However, it may provide important information or evidence for elucidating the competition of recognizing cell-bound receptors among various lipoproteins. AFM detection of interaction forces between lipoproteins and free/cell-bound receptors may provide further important information although it is very challenging. More in-depth studies will be needed.

## Conclusions

Taken together, the morphological and receptor-binding properties of three native (HDL, LDL, and VLDL) and two modified (oxLDL and acLDL) lipoproteins were detected and compared by AFM at the nanoscale under a physiological condition (in PBS). According to our knowledge, it is the first time that the sizes of modified lipoproteins and the receptor-binding abilities of lipoproteins were evaluated at the nanoscale by AFM. The data may provide important information for better understanding of the structures and functions of various lipoproteins. The data also certify the usefulness of AFM for obtaining more important information (e.g. the receptor-lipoprotein binding ability) besides the sizes of lipoproteins.

## Abbreviations

Ac-LDL: acetylated low-density lipoprotein
AFM: atomic force microscopy
BSA: bovine serum albumin
FWHM: full width at half maximum
HDL: high-density lipoprotein
LDL: low-density lipoprotein
Ox-LDL: oxidized low-density lipoprotein
VLDL: very low-density lipoprotein
